# IgG subclass antibodies to human and bacterial HSP60 are not associated with disease activity and progression over time in axial spondyloarthritis

**DOI:** 10.1186/ar4234

**Published:** 2013-05-25

**Authors:** Thomas Gelsing Carlsen, Astrid Hjelholt, Anne Grethe Jurik, Berit Schiøttz-Christensen, Anna Zejden, Gunna Christiansen, Bent Deleuran, Svend Birkelund

**Affiliations:** 1 Department of Health Science and Technology, Aalborg University, Fredrik Bajers Vej 3b, 9220 Aalborg Ø, Denmark; 2 Department of Biomedicine--Medical Microbiology & Immunology, Aarhus University, The Bartholin Building, Wilhelm Meyer's Allé 1240, 8000 Aarhus C, Denmark; 3 Department of Radiology, Aarhus University Hospital, Nørrebrogade 44, 8000 Aarhus C, Denmark; 4 Aarhus Clinic for Rheumatic Diseases, Clemens Torv 17, 8000 Aarhus C, Denmark; 5 Department of Rheumatology, Aarhus University Hospital, Nørrebrogade 44, 8000 Aarhus C, Denmark; 6 Loke Diagnostics, Sindalsvej 17, 8240 Risskov, Denmark

**Keywords:** Spondyloarthritis, heat shock protein 60, HSP60, HSPD1, HLA-B27, IgG subclass

## Abstract

**Introduction:**

Spondyloarthritis (SpA), an interrelated group of rheumatic diseases, has been suggested to be triggered by bacterial infections prior to the development of an autoimmune response that causes inflammation of the spinal and peripheral joints. Because human heat shock protein 60 (HSP60), recently renamed HSPD1, and bacterial HSP60 are highly homologous, immunological cross-reactivity has been proposed as a mechanism of disease initiation. However, previous investigations of the humoral immune response to HSP60 in SpA patients have lacked determination of immunoglobulin G (IgG) subclasses and patient follow-up. In this study, we have focused on these parameters in a cohort of axial SpA patients with a well-established set of clinical characteristics, including MRI changes and human leukocyte antigen B27.

**Methods:**

IgG subclass antibodies (IgG1, IgG2, IgG3 and IgG4) against recombinant HSP60 of three reactive arthritis-related bacteria; human HSP60; and the microorganisms *Chlamydia trachomatis *and *C. pneumoniae *were determined by ELISA. Serum samples collected from 2004 to 2006 and in 2010 and 2011 from 39 axial SpA patients were analyzed and compared with samples from 39 healthy controls. The Mann-Whitney *U *test and Wilcoxon matched pairs test were used to compare the antibody levels in different and paired groups, respectively. *P *< 0.01 was considered significant. The Spearman nonparametric correlation was used to determine correlation between antibody levels and between antibody levels and the disease parameters.

**Results:**

Elevated levels of IgG1 and IgG3 to human HSP60 and IgG1 to HSP60 of *Salmonella enterica *Enteritidis were observed in SpA patients compared with healthy controls at both time points. The antibody levels were almost constant over time for IgG1, whereas high levels of IgG3 to human HSP60 tended to decrease over time. The antibody response to human HSP60 was predominantly of the IgG3 subclass, and patients with high levels of IgG3 to this antigen had low levels of IgG1, indicating an inverse association. Different IgG subclasses were produced against bacterial and human HSP60 in the same serum sample, IgG1 and IgG3, respectively, indicating that there was no cross-reaction.

**Conclusions:**

A significant association was observed between axial SpA and the presence of IgG1/IgG3 antibodies to human HSP60 and of IgG1 to *S. enterica *Enteritidis and *C. trachomatis*. Generation of antibodies to human HSP60 was independent of the presence of antibodies to bacterial HSP60. No association was observed between clinical and MRI changes with antibodies over time. Altogether, such antibodies do not reflect the disease activity in these patients.

This study has been approved by the Regional Research Ethics Committee of Central Jutland, Denmark. Trial registration numbers: 20050046 and 20100083

## Introduction

For more than two decades, heat shock proteins (HSPs) have been known for their phylogenetically conserved composition and immune-modulating activities [1]. They are ubiquitous in cellular life and exist in both eukaryotic and prokaryotic cells, where their major role is to act as molecular chaperones. Over the past few years, it has become evident that, in addition to their function as intracellular chaperones, HSPs are also found in the cell membrane and outside the cell, presumably acting as indicators of the stress conditions and activating other cells, particularly cells of the immune system [2]. As a response to stress conditions, their expression level is increased to prevent aggregation of misfolded proteins [3]. Such conditions are prevalent during intracellular bacterial infections, where the highly conserved HSPs from the 60 kDa family (HSP60; also known as GroEL) act as potent stimulators of both the innate and adaptive immune systems [4].

Some rheumatic diseases, such as reactive arthritis (ReA) and Lyme disease, are associated with bacterial infections. In connection with this, it has been suggested that bacteria-related autoimmunity may be an important factor in the etiology of such diseases [5]. One hypothesis to explain the pathogenic mechanism after a bacterial infection is molecular mimicry, that is, sharing of linear or conformational epitopes common to microbial antigens and host cell molecules, giving rise to an inappropriate immune response [6]. Human HSP60, recently renamed HSPD1 in a proposed new nomenclature [7], shares more than 50% of its sequence with bacterial HSP60, and, consequently, antibody and T-cell recognition of human HSP60 have been investigated extensively in a number of studies. Such antibodies are found both in patients with different inflammatory diseases and in healthy individuals, indicating that shared epitopes between bacterial and human HSP60 may exist [8-13]. However, studies have yet to determine the pathogenic role for such autoantibodies and whether these are in fact cross-reactive [14].

SpA comprises a heterogeneous group of immune-mediated inflammatory diseases, which includes the bacteria-triggered (that is, *Chlamydia trachomatis, Salmonella, Yersenia *and *Campylobacter jejuni*) disease reactive arthritis (ReA). Even though a link to an antecedent infection is less clear for the other forms of SpA diseases [15], the group shares clinical, pathological and genetic features, and especially the link to human leukocyte antigen B27 (HLA-B27) is well-documented [16]. However, knowledge about how HLA-B27 may contribute to disease development is missing [17]. Moreover, the frequency of the HLA-B27 genotype varies in the different SpA diseases [16].

The most significant results revealing the importance of the host-pathogen interaction in SpA disease came from studies done in rodents [18]. Such studies demonstrated that HLA-B27-transgenic rats do not develop inflammatory pathology in the intestine or the joints as long as they are kept in a germ-free environment [19]. Human studies include transfection of human cell lines with HLA-B27 and investigation of a possible interaction with pathogenic arthritis-related bacteria through changes in cytokine profiles, T-cell responses or HLA-B27 expression. However, results from these studies seem to differ, depending on the investigated pathogens [20-22]. As in ReA, autoimmunity against HSP60 has also been speculated as a trigger mechanism in SpA disease development, but such studies are few, and often the patient material was split up into disease subgroups [13,23,24]. In addition, existing serological analyses targeting arthritis-related bacterial HSP60 in SpA patients were without subclass specificity and follow-up [13,23,24]. Furthermore, in these studies, it was not clear whether the purification method of the HSP60 antigens retained the *in vivo *conformation. Improvements of these parameters would increase not only the specificity in the IgG response but also the analysis of cross-reactivity and the ability to determine whether such antibodies are of importance in SpA pathogenesis. Recently, a serology study on females diagnosed with tubal factor infertility (TFI) was performed, and it was shown by ELISA that IgG antibodies to *C. trachomatis *HSP60 were of the IgG1 and IgG3 subclass and that no antibodies against human HSP60 could be detected [25]. In that study, it was demonstrated that determination of IgG1 subclass antibodies to chlamydial HSP60 increased the diagnostic value of the ELISA in identifying TFI.

Therefore, we adopted this approach and performed a study to analyze the quantified serum levels of IgG subclass antibodies to native bacterial and human HSP60 in a cohort of well-characterized SpA patients compared with an age- and gender-matched control group. We evaluated the association between antibody levels and disease severity assessed by clinical scoring comprising the Bath Ankylosing Spondylitis Metrology Index (BASMI), Bath Ankylosing Spondylitis Functional Index (BASFI), Bath Ankylosing Spondylitis Disease Activity Index (BASDAI) and MRI changes in the spine and sacroiliac joints (SIJs) in addition to HLA-B27 status.

## Materials and methods

### Patients and healthy controls

The study included two serum samples from SpA patients (*n *= 39) with symptoms restricted to the axial skeleton. Serum samples were collected from 2004 through 2006 and again in 2010 and 2011. At each point in time, clinical, radiological and MRI measurements were performed [26,27]. The clinical scorings comprised BASMI, BASFI and BASDAI. In addition C-reactive protein (CRP) concentration and the HLA-B27 status were determined. At the time of study entry, the examination included serum sampling and MRI of the SIJ and the spine, which was scored according to the Danish system [26,27]. All patients met the European Spondyloarthropathy Study Group (ESSG) and the Assessment of SpondyloArthritis international Society (ASAS) criteria [28,29]. Serum samples were collected from age- and gender-matched healthy volunteers (*n *= 39) in 2010 and served as controls. The characteristics of the patients and healthy controls are given in Table 1.

**Table 1:** Characteristics of SpA patients and healthy controls

Characteristics	SpA patients (*n *= 39)		HCs (*n *= 39)
	**2006**	**2010**	
		
Mean age, yr	38 (23-49)	42 (27-53)	37 (23-58)
Gender, % females	56	-	64
HLA-B27-positive, %	54	-	ND
Disease duration, mean, years	8 (3-19)	ND	0
Anti-TNF treatment	3	6	
Average MRI activity score			
SIJ	4.8 (2.0-2.1)	2.5 (0.4-6.3)	ND
Spine	2.0 (0.0-3.3)	1.0 (0.0-2.0)	ND
Average MRI chronicity score			
SIJ	12.0 (3.0-27.8)	17.8 (3.0-31.5)	ND
Spine	0.0 (0.0-4.0)	1.0 (0.0-5.5)	ND
CRP (normal <8 mg/L)	1.89 (1.26-3.36)	1.3 (0.5-2.55)	ND
BASDAI	32 (14-53)	28 (15-50)	ND
BASMI	0 (0-40)	0 (0-10)	ND
BASFI	18 (6-33)	14 (8-34)	ND

SpA: spondyloarthritis, HC: healthy control, ND: not done, NT: no treatment, CRP: C-reactive protein, BASDAI: Bath Ankylosing Spondylitis Disease Activity Index, BASMI: Bath Ankylosing Spondylitis Metrology Index, BASFI: Bath Ankylosing Functional Index, MRI: magnetic resonance imaging, SIJ: sacroiliac joint. Data are shown as medians (if not stated otherwise) with interquatile ranges (IQRs).

The patients were enrolled from the outpatient clinic at Aarhus University Hospital after they gave their informed, written consent according to the Danish Data Protection Agency, the Regional Ethics Committee of Central Jutland, Denmark (project numbers 20050046 and 20100083), and the Declaration of Helsinki.

### Characteristics of the patients

There was no significant difference between SpA and the control group regarding age and gender, whereas the number of HLA-B27-positive persons was higher in the patient group (54%) than in the control group (8% in Caucasians) (Table 1) [30]. The duration of the disease was, on average, 8 years when the first blood sample was drawn. Most of the patients (*n *= 36) did not receive any treatment at the time of enrollment in the study (Table 1). C-reactive protein (CRP) was within the normal range at both time points (Table 1). All patients showed signs of inflammation on MRI, with decreased activity over time.

### Cloning and purification of recombinant HSP60

*Escherichia coli *clones containing the genes encoding full-length human HSP60 and *C. trachomatis *HSP60 were obtained from Loke Diagnostics (Risskov, Denmark). *C. jejuni *and *S. enterica *Enteritidis HSP60 genes were cloned in pET30ek-LIC vector (Invitrogen, Carlsbad, CA). The *C. jejuni *HSP60 gene was amplified with the forward primer GACGACGACAAGATGGCAAAAGAAATTATTTTTTCAGATGAAGC and reverse primer GAGGAGAAGCCCGGTTTACATCATTCCTCCCATGCC. For the *S. enterica *Enteritidis HSP60 gene, the forward primer GACGACGACAAGATGGCAGCTAAAGACGTAA-AATTCGG and reverse primer GAGGAGAAGCCCGGTTTACATCATGCCGCCC were used. The PCR products were cloned into pET30ek-LIC by ligase-independent cloning according to the manufacturer's instructions (Novagen; Merck KGaA, Darmstadt, Germany).

The HSP60 proteins were expressed in *E. coli *BL21 (DE3) using 1 mM isopropyl-β-D-thiogalactopyranoside (IPGT) for 2 h. A sample was removed from a 500-ml bacterial culture before and after induction and analyzed by sodium dodecyl sulfate-polyacrylamide gel electrophoresis (SDS-PAGE) [31].

The recombinant HSP60 proteins for ELISA were purified by Ni^2+ ^affinity chromatography under non-denaturing condition using an imidazole step gradient for passing through a Hi-Trap column (GE Healthcare Bio-Sciences AB, Uppsala, Sweden) as described by Schmitt *et al. *[32]. Fractions that contained protein were pooled together into separate Spectra/Por 1 membrane bags (Spectrum Laboratories Europe, Breda, The Netherlands) and dialyzed against PBS with EDTA (1 mM) for 20 h. Endotoxin levels of purified protein were measured using Limulus Amebocyte Lysate (LAL) QCL-1000 according to 'the manufacturer's instructions (Lonza, Basel, Switzerland). None of the purified samples exceeded 5 EU/mg protein.

The purified HSP60 proteins and the total protein content of induced protein from *E. coli *BL21 (DE3) were analyzed by 10% SDS-PAGE [31]. To determine the molecular size of the recombinant protein, Mark 12 (Invitrogen Life Technologies, Carlsbad, CA, USA) was used.

### Enzyme-linked immunosorbent assay (ELISA)

The prevalence of antibodies was determined by ELISA using subclass-specific secondary antibodies. The ELISA was performed as described by Hjelholt *et al. *[25] and Drasbek *et al. *[33]. ELISA plates were coated with 4 μg/ml human HSP60, *C. trachomatis *HSP60, *C. jejuni *HSP60 or *S. enterica *Enteritidis HSP60. The human serum samples were diluted 1:50 in BAC-DIL (medac, Hamburg, Germany) before use. The secondary anti-human IgG antibodies used were conjugated horseradish peroxidase (HRP), sheep anti-human IgG1, IgG2, IgG3 and IgG4 (Binding Site, Birmingham, UK), diluted in BAC-DIL. The dilutions were chosen so that the 450-nm optical density (OD_450_) levels were localized on the linear portion of the standard curve. For quantification of IgG subclass antibodies, NUNC MaxiSorp plates were coated with dilution series of native IgG1, IgG2, IgG3 and IgG4 from human myeloma plasma (EMD Biosciences, San Diego, CA, USA) in carbonate coating buffer (50 mM NaHCO_3_, pH 9.6). The ELISA was performed using the respective secondary antibodies.

All samples were measured in duplicate. To diminish intra-assay variation, serum samples from each patient were measured next to each other on the ELISA plate. Each plate had a positive and negative control included to diminish interassay variation. In this study, intra- and interassay variability was less than 10% and 5%, respectively. OD was read with a Tecan Sunrise reader at 450 nm and 620 nm as a reference length, and the ELISA results were analyzed using Magellan Data Analysis Software (Tecan, Männedorf, Switzerland). The *C. trachomatis*-IgG-ELISA-plus plate (medac) [34] were used for the major outer membrane protein peptide-based analysis, and the *C. pneumoniae*-IgG-ELISA-plus plate (medac) was used to determine IgG subclass antibodies to *C. pneumoniae *(medac).

### Statistical analysis

The data were analyzed using GraphPad Prism version 5.0a software for Mac OS × (GraphPad Software Inc., La Jolla, CA, USA). The Shapiro-Wilk normality test was performed to reject normally distributed data (α = 0.05, *P *< 0.0001). The Mann-Whitney rank-sum test was used to compare differences between antibody levels in different groups. The Wilcoxon matched-pairs test for paired samples was used to compare differences between antibody levels in paired samples. The Spearman nonparametric correlation coefficient was used to analyze the correlation between antibody levels, as well as between antibody levels and the score values from BASDAI, BASFI, BASMI and for activity and chronic SpA changes by MRI. *P *< 0.01 was considered statistically significant. The confidence interval used was 99%.

## Results

### Purification of recombinant HSP60 and optimization of ELISA

**Figure 1:**
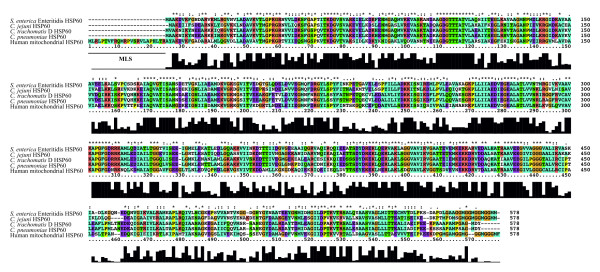
**Multiple sequence alignment of bacterial and human HSP60**. A multiple sequence alignment was created with ClustalX 2.0.12 software to compare the amino acid sequences of bacterial HSP60 (*Salmonella enterica *Enteritidis [GenBank:YP_002246130.1], *Campylobacter jejuni *[GenBank:CAA73778.1], *C. trachomatis *[GenBank:ADI51797.1]) and *C. pneumoniae *[GenBank:P31681.2]) and human HSP60 [GenBank:NP_955472.1]. The bar diagram illustrates areas of high sequence consensus. MLS: mitochondrial leader sequence.

**Figure 2:**
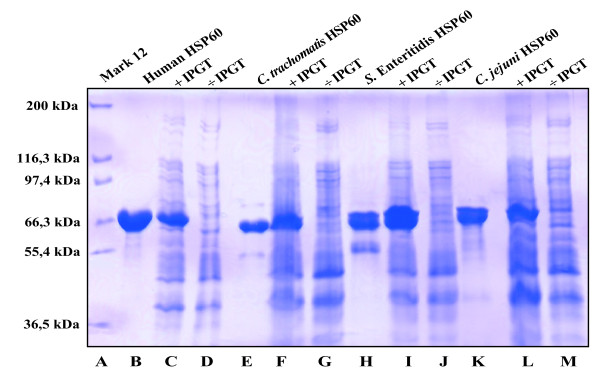
**SDS-PAGE gel of bacterial and human HSP60**. Size and purity of each recombinant protein were evaluated using 10% SDS-PAGE gel. The different HSP60 proteins for ELISA were purified using a Ni^+ ^column and imidazole as a competing reagent. Vector transformation was confirmed by isopropyl-β-D-thiogalactopyranoside (IPGT) induction. Lane *A*: Molecular weight marker. Lanes *B, E, H *and *K*: Purified HSP60. Lanes *C *and *D, F *and *G, I *and *J *and *L *and *M*: Total protein content from *Escherichia coli *strain BL21 (DE3) with (+) and without (-) IPGT induction.

A multiple sequence alignment of the linear amino acid sequence of the HSP60 proteins from *S. enterica *Enteritidis, *C. jejuni, C. trachomatis *and *C. pneumoniae *and from human HSP60 are shown in Figure 1. Stars above and bars underneath the sequences mark identical amino acids. *C. trachomatis *HSP60 shares more than 90% identity with *Chlamydia *spp., 60% identity with other bacteria and 50% identity with human HSP60 [35]. This comparison, which reflects the linear sequence, is important because measured antibodies targeting bacterial HSP60 have been proposed to cross-react with the human homolog and elicit the autoimmune reaction seen in bacteria-triggered arthritis [9,10,13]. To increase antibody specificity and assay sensitivity, we purified the HSP60 antigens in their native form, leaving out denaturants in the preparation of the recombinant protein or as part of the elution buffer [32]. Instead, the elution buffer consisted of imidazole at nondenaturing concentrations, which is passed through the column as a step gradient. The excess of imidazole displaces the His tag from the nickel column, freeing the His-tagged proteins. Induction and purity of the eluted HSP60 protein was analyzed by SDS-PAGE, as shown in Figure 2. The total protein content from the uninduced (-IPTG; lanes D, G, J and M) and induced (+IPTG; lanes C, F, I and L) samples was analyzed by SDS-PAGE next to the purified HSP60 proteins (lanes B, E, H and K). Induced and purified proteins were of similar size (Figure 2).

**Figure 3:**
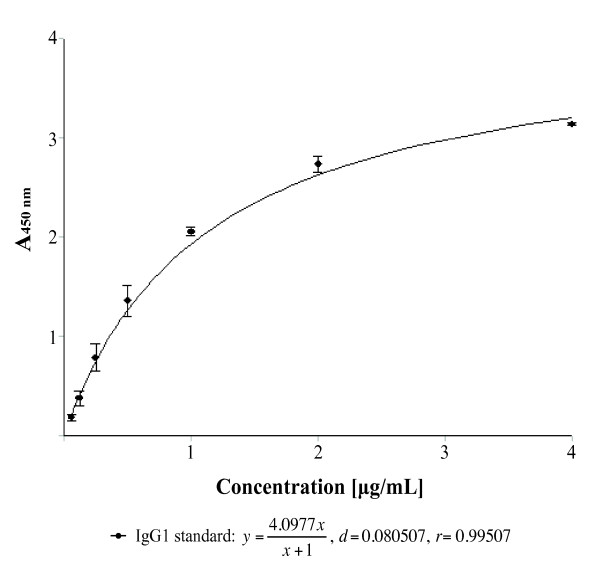
**Standard curve for IgG1**. A standard curve was made using Magellan Data Analysis Software to quantify the A_450 nm _values from ELISA. An ELISA plate was coated with a known serial dilution (0.0625-4 µg/ml) of native IgG1 subclass from human myeloma plasma before incubating wells with HRP-conjugated secondary antibody. Correlation between A_450 nm _values and concentration was fitted by the regression output (*r *= 0.99507).

The optimization of the ELISA was done to obtain the optimal dynamic range of the measured antibodies using a single dilution of the serum samples (1:50). To avoid deficiency in epitopes, the antigen was used in a high coating concentration (4 µg/ml). A strongly seropositive serum sample was diluted 1:50 and evaluated with different concentrations of each enzyme-conjugated IgG subclass antibody. The concentration of the secondary antibody was determined from the dilution used to obtain an OD value of 2 for this serum sample (1:10,000 for IgG1). A standard curve for IgG1 was generated by coating an ELISA plate with dilutions of myeloma IgG1 as the antigen and reacting it with HRP-conjugated goat anti-human IgG1 diluted 1:10,000 as shown in Figure 3. Similarly, standard curves were made for the other subclass antibodies. Such curves were used to determine the immunoglobulin concentration (µg/ml) from the OD values determined by ELISA.

### Clinical characteristics of the SpA patients and healthy controls

During the observation period, there was no significant overall change in CRP, BASDAI, BASMI or BASFI; however, a tendency toward an increase in the BASDAI, BASFI and BASMI score was observed. During the period, 11 patients decreased and 22 patients increased in BASDAI, 13 patients decreased and 19 patients increased in BASFI, and 3 patients decreased and 10 patients increased in BASMI. The MRI activity score for SIJ decreased significantly (*P *= 0.0001), whereas the spinal MRI activity score did not change. As expected, a slight but nonsignificant increase was present in the MRI evaluation of both spine and SIJ regarding chronicity. There was a general tendency toward low disease activity in patients at both time points. Six patients received treatment with anti-TNF during the period of observation (Table 1).

### IgG1 and IgG3 antibodies to human and bacterial HSP60 in SpA patients and healthy controls

**Figure 4:**
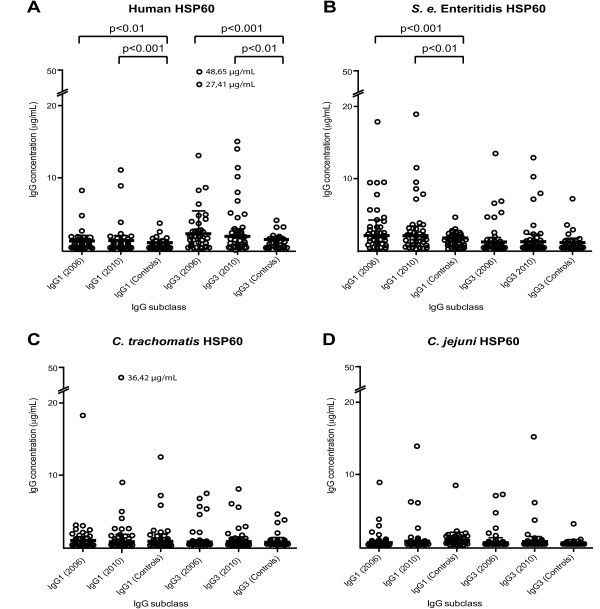
**Antibody levels to human and bacterial HSP60 in the SpA cohort group and the control group**. Serum levels (µg/ml) of IgG1 and IgG3 antibodies against **(A) **human HSP60, **(B) ***Salmonella enterica *Enteritidis, **(C) ***Chlamydia trachomatis *HSP60 and **(D) ***Campylobacter jejuni *HSP60 in the SpA cohort group and the control group. The bars represent the medians with IQRs. The scale on the *y*-axis has been modified after 20 µg/ml to include outliers and increase resolution. Statistical analyses of differences between groups were done with the nonparametric Mann-Whitney rank-sum test. Only probabilities <0.01 were considered significant and are highlighted in the figure.

Individual levels of the IgG subclass antibodies to human HSP60 (Figure 4A) and HSP60 of *S. enterica *Enteritidis (Figure 4B), *C. trachomatis *(Figure 4C) and *C. jejuni *(Figure 4D) in SpA patients and controls are shown in Figure 4. Although IgG1 and IgG3 antibodies were detected in serum samples of both SpA patients and controls, no IgG2 or IgG4 antibodies were detected for any of the four HSP60 antigens.

The levels of anti-human HSP60 for IgG1 and IgG3 are shown in Table 2. In SpA patients, these levels are significantly higher than those of healthy controls (Table 3). Elevated levels of IgG1 against *S. enterica *Enteritidis HSP60 were found in SpA patients compared with healthy controls (Table 3). There were no significant differences in IgG1 and IgG3 to *C. trachomatis *HSP60 and *C. jejuni *HSP60 between SpA patients and healthy controls (Table 3). Levels of IgG3 against human HSP60 were higher than levels of IgG1 at both time points (*P *< 0.001, Table 2). The highest antibody levels for IgG3 against human HSP60 were measured in 2006 (Figure 4A); however, no significant difference in IgG1 and IgG3 levels was found between paired samples (2006 through 2010), respectively, for any of the investigated HSP60 antigens (Figure 4). When the patients were divided into two groups according to the HLA-B27 genotype, no significant differences in antibody levels were observed between HLA-B27-positive and HLA-B27-negative patients for any of the investigated HSP60 antigens.

**Table 2:** Medians and interquatile ranges (IQRs) of IgG1 and IgG3 antibody levels (μg/mL) against six different antigens in the SpA group

Antigen	Median (IQR)			
	**2006**		**2010**	
	
	**IgG1**	**IgG3**	**IgG1**	**IgG3**
	
Human HSP60	2.046 (1.896-2.345)	2.689 (2.476-4.827)	2.131 (1.936-2.503)	2.583 (2.455-3.475)
*Salmonella enterica *Enteritidis HSP60	2.635 (2.109-4.300)	2.514 (2.443-2.799)	2.660 (2.240-3.681)	2.534 (2.452-3.142)
*Chlamydia trachomatis *HSP60	2.123 (1.939-2.694)	2.522 (2.453-2.726)	2.096 (1.957-2.747)	2.631 (2.472-2.960)
*Campylobacter jejuni *HSP60	2.459 (2.347-2.640)	2.447 (2.403-2.628)	2.496 (2.395-2.622)	2.462 (2.409-2.684)
*Chlamydia pneumoniae*	7.965 (2.807-24.56)	2.397 (2.397-2.730)	8.953 (2.839-21.75)	2.450 (2.397-2.777)
*Chlamydia trachomatis*	2.082 (2.052-2.174)	2.416 (2.406-2.473)	2.103 (2.065-2.216)	2.421 (2.410-2.491)

**Table 3:** Statistical differences in IgG1 or IgG3 levels between SpA patients (*n *= 39) and healthy controls (*n *= 39) against six different antigens

Antigen	IgG1		IgG3	
	**2006**	**2010**	**2006**	**2010**
	
Human HSP60	0.0082**	0.0004***	0.0001***	0.0022**
*Salmonella enterica *Enteritidis HSP60	0.0008***	0.0002***	0.8455	0.2782
*Chlamydia trachomatis *HSP60	0.9164	0.7453	0.0932	0.8338
*Campylobacter jejuni *HSP60	0.0451	0.1109	0.9482	0.5587
*Chlamydia pneumoniae*	0.3738	0.4211	0.0892	0.3748
*Chlamydia trachomatis*	0.0089**	0.0786	0.2693	0.4010

SpA: spondyloarthritis, HC: healthy control. The Mann-Whitney rank-sum test was used to compare the SpA cohort group (2006 and 2010) with healthy controls. Ig, immunoglobulin. ***P < 0. 001. **P < 0.01.

### IgG1 and IgG3 antibodies to *C. pneumoniae, C. trachomatis *and HSP60 from *C. trachomatis*

**Figure 5:**
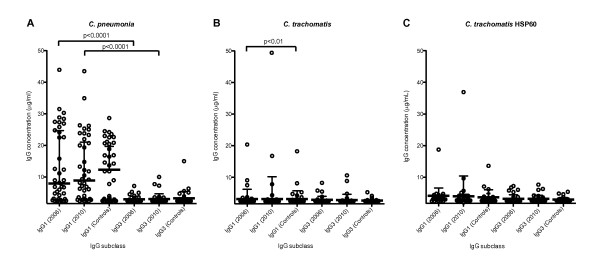
**Antibody levels to *Chlamydia pneumoniae, *C**. *trachomatis and C. trachomatis *HSP60 in the SpA cohort and the control group. Serum levels (µg/ml) of IgG1 and IgG3 against **(A) ***C. pneumonia*, **(B) ***C. trachomatis *and **(C) **HSP60 of *C. trachomatis *in the SpA cohort and healthy controls. The bars represent the medians with IQRs. Only probabilities <0.01 were considered significant and are highlighted in the figure.

To determine whether antibodies to *C. pneumoniae *or antibodies to *C. trachomatis *were associated with antibodies to HSP60 from *C. trachomatis*, individual levels of IgG antibodies (IgG1 and IgG3) against *C. pneumoniae, C. trachomatis *and *C. trachomatis *HSP60 were determined in SpA patients and healthy controls (Figure 5). A large proportion of both SpA patients (IgG1 = 84.6%, detection limit OD = 0.1) and controls (IgG1 = 76.9%, detection limit OD = 0.1) had IgG1 antibodies against *C. pneumoniae *(Figure 5A) [36]. Levels of IgG1 antibodies against *C. pneumoniae *(Table 2) were significantly higher than levels of IgG3 antibodies (Figure 5A, P < 0.0001). Low levels of both IgG1 and IgG3 antibodies were found against *C. trachomatis *(Figure 5B) and *C. trachomatis *HSP60 (Figure 5C), and only a few serum samples were positive (Figure 5B). However, a significant difference between SpA patients and controls was observed for IgG1 antibodies against *C. trachomatis *in 2006 (Figure 5B, P < 0.01; see also Table 3). In one serum sample, high levels of IgG1 antibodies against *C. trachomatis *were seen both in 2006 (20.1 μg/ml) and in 2010 (49.7 μg/ml). This serum sample also had IgG1 antibodies to *C. trachomatis *HSP60 (2006 = 17.7 μg/ml and 2010 = 36.4 μg/ml) and to *C. pneumoniae *(2006 = 30 μg/ml and 2010 = 23.7 μg/ml), indicating an ongoing or recent *Chlamydia *infection.

### IgG1 and IgG3 antibodies to human HSP60 in SpA patients

**Figure 6:**
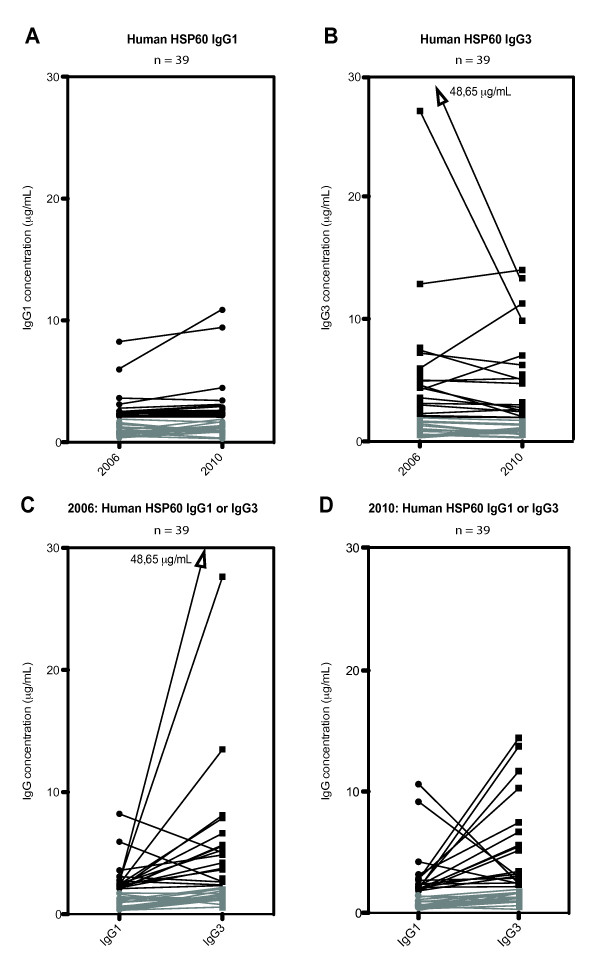
**Change in antibody levels against human HSP60 in sera from SpA patients**. Change in serum levels (µg/ml) and subclass specificity of IgG1 (circle) and IgG3 (square) against human HSP60 (*n *= 39). A line links each serum value from 2006 with that of 2010. **(A) **Change in serum levels of IgG1 from 2006 through 2010. **(B) **Change in serum levels of IgG3 from 2006 through 2010. **(C) **Antibody level between IgG1 and IgG3 in 2006. (D) Antibody level between IgG1 and IgG3 in 2010. Serum levels below 2 μg/ml are shown in gray.

To visualize the course of anti-human HSP60 IgG1 and IgG3 over time, subclass antibody levels for the SpA group (*n *= 39) were plotted and a line was drawn connecting the serum samples from 2006 and 2010, respectively (Figures 6A and 6B). The antibody levels of IgG1 (Figure 6A) and IgG3 (Figure 6B) showed no significant change from 2006 to 2010. Levels of IgG1 were mainly unchanged (Figure 6A), whereas IgG3 showed both increases and decreases (Figure 6B). Two of the serum samples had high levels of IgG3 (27.41 μg/ml and 48.65 μg/ml, respectively) in 2006, and there was a decrease from 2006 to 2010 (Figure 6B).

A comparison of the levels of IgG1 with those of IgG3 in the same serum sample (*n *= 39) against human HSP60 from 2006 showed a trend toward an inverse correlation (r = -0.2). In the serum samples taken in 2010, a similar inverse correlation (r = -0.054) was observed, and it was more distinct at high IgG subclass levels (Figures 6C and 6D).

### Investigation of cross-reactive antibodies in SpA patients

**Figure 7:**
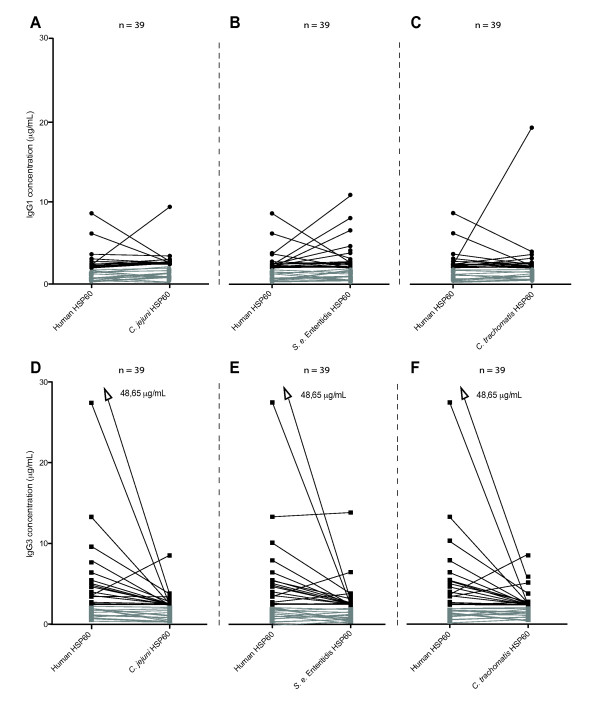
**Comparison of antibody levels against human and bacterial HSP60 in SpA patients**. Antibody levels in serum samples from 2006 against human HSP60 (*n *= 39) were compared groupwise with IgG1 **(A)**, **(B) **and **(C) **and IgG3 **(D)**, **(E) **and **(F) **antibodies against bacterial HSP60. Lines are drawn between antibody levels for human HSP60 and HSP60 of *Salmonella enterica *Enteritidis, *Chlamydia trachomatis *and *Campylobacter jejuni *HSP60 for each patient. Serum levels below 2 μg/ml are shown in gray.

To compare the IgG subclass levels, lines were drawn between IgG1 of human HSP60 and IgG1 of HSP60 from *S. enterica *Enteritidis, *C. trachomatis *and *C. jejuni *(Figures 7A-7C). Samples with high levels of IgG1 antibodies against human HSP60 tended to show low levels of IgG1 against bacterial HSP60 and vice versa (Figures 7A-7C). A similar trend was observed for IgG3 antibodies (Figures 7D-7F), with the exception of one serum sample positive for *S. enterica *Enteritidis (Figure 7E). This observation suggests that antibodies to human HSP60 develop independently of antibodies to bacterial HSP60, even though antibodies to a single bacterial HSP60 frequently were seen in serum samples from these individual patients. No significant correlation was observed between the two subclass antibodies against any of the bacterial HSP60 or human HSP60 (*P *> 0.01), which is in agreement with the negative correlation observed between IgG1 and IgG3 antibodies against human HSP60, and thus we observed no cross-reaction between human and microbial HSP60. However, correlations between IgG1 and IgG3, respectively, against the three bacterial HSP60 were observed (data not shown). The strongest correlation was between IgG1 against HSP60 from *S. enterica *Enteritidis and *C. jejuni *in 2006 (*r *= 0.7, *P *< 0.001), which is in agreement with their higher amino acid sequence identity (Figure 1).

### IgG1 and IgG3 anti-HSP60 related to disease severity

No significant correlation was found between the presence of antibodies to either human or bacterial HSP60 and age, sex, treatment, CRP, MRI scores, HLA-B27 and any of the BAS indexes (*P *> 0.01). The change in antibody levels compared with change in BAS indexes and MRI change from 2006 to 2010 also did not show any correlation (*P *> 0.01).

## Discussion

In the present study, we investigated the hypothesis of molecular mimicry as a pathogenic trigger in axial SpA. Potential cross-reaction regions were visualized in an alignment of the amino acid sequences of human and bacterial HSP60, which showed five regions with five to eight identical amino acid residues for all of the investigated HSP60 proteins, indicating the presence of potential epitopes (Figure 1). However, these protein regions may not represent epitopes for infection-induced antibodies, as most antibodies recognize conformational epitopes presented in the three-dimensional shape of the protein [37], and, even if cross-reactive antibodies were present, it would not prove causation of disease. Therefore, due to the lack of similar studies that include analysis of IgG subclass response to bacterial and human HSP60, this hypothesis is a target for further analysis [11,23,24,38].

In our study, we found elevated levels of both IgG1 and IgG3 antibodies to human HSP60, indicating an increased autoimmune activity in the 39 axial SpA patients (Figure 4A). We found no correlations of IgG antibodies between bacterial and human HSP60, and there was no general elevation in levels of antibodies to bacterial HSP60 (Figures 4B-4D). In the present study, generation of antibodies to human HSP60 was independent of the presence of antibodies to bacterial HSP60, and cross-reactivity was not found (Figure 7). In addition, different subclasses (that is, IgG1 and IgG3) were found to be predominant in the antibody response to bacterial and human HSP60, thus strengthening the ability of the humoral immune response to maintain its specificity, even between similar antigenic molecules.

A significant elevation in levels of IgG1 antibodies against HSP60 from *S. enterica *Enteritidis, but not from C. *jejuni *and *C. trachomatis*, was also observed in the group of SpA patients compared with the control group (Figure 4). These findings suggest that more SpA patients have had an infection with *Salmonella *than with an infection with *C. trachomatis *or *C. jejuni*. They are in agreement with a high incidence in *S. enterica *Enteritidis infections in Denmark that increased from 1997 to 1999 [39]. The observation that a higher number of patients are positive for *S. enterica *Enteritidis than for *C. trachomatis *(Figures 4B, 5B and 5C) could be explained by the overweight of females (56%, Table 1), as postdysentery ReA is equally common in males and females, whereas postchlamydial ReA is much more common in males [40].

To investigate the IgG subclass response against bacterial antigens, we determined the IgG subclass response to *C. pneumoniae *and *C. trachomatis *(Figure 5). We found that the IgG antibody response against *C. pneumoniae *was of the IgG1 subclass (Figure 5A). Thus, the production of IgG3 antibodies was not a general response to bacterial antigens in SpA patients. Furthermore, as recently suggested by Carter *et al*., a previous infection with *C. pneumoniae *may be associated with SpA [41]. We therefore evaluated the association between the SpA disease and levels of antibodies to *C. pneumoniae *[34]. However, there was no statistical difference in the IgG1 antibody levels of SpA patients and controls. In 1999, Hannu *et al*. used microimmunofluorescence microscopy to detect antibodies to *C. pneumoniae*, and they found that 4 (approximately 10%) of 35 patients with ReA were highly positive. Of these four patients, three had had lower respiratory tract infection prior to the development of ReA, indicating a possible trigger of the disease [42]. We found that 84% of the SpA patients and 76.9% of controls had IgG1 antibodies to *C. pneumoniae*, but such antibodies were not correlated with the presence of chlamydial HSP60 antibodies. This finding indicates that a *C. pneumoniae *infection does not trigger production of antibodies to chlamydial HSP60, even though HSP60 of *C. pneumoniae *is highly similar to HSP60 of *C. trachomatis *(90%, Figure 1). This is in contrast to what was found when serum from TFI patients was analyzed for the presence of subclass antibodies [25]. Antibodies to both *C. trachomatis *and to *C. trachomatis *HSP60 were found in serum from TFI patients. However, although antibodies to chlamydial HSP60 were primarily IgG1, antibodies to *C. trachomatis *were predominantly IgG3. None of the TFI patients had antibodies to human HSP60, indicating that in this study no cross-reactive antibodies between human and bacterial HSP60 were found [25].

IgG1 and IgG3 have similar properties in terms of activating complement and binding of Fc receptors [43]. This may explain why they are often induced together, indicating a shared switch mechanism (via IL-21 stimulation), as reported by Péne *et al. *[44]. However, on the basis of the results of the present study, IgG3 was the predominant antibody subclass to human HSP60. Furthermore, a trend toward an inverse association between IgG1 and IgG3 antibodies to this antigen was seen (Figures 6C and 6D), indicating the presence of a fine-tuning mechanism, recently suggested to be IL-4 [45]. A study similar to ours showed the same distribution of the IgG subclass response to a self-antigen, peptidylarginine deiminase 4 (PADI4), in patients with rheumatoid arthritis (RA) [46]. However, in this study, the dominant subclass was IgG1 and not IgG3. The structure of the two molecules differs in that IgG3 is more flexible due to its longer hinge region [47]. They also differ in half-life (1 wk for IgG3 compared to 3 wk for IgG1), and their concentrations in serum vary (65% for IgG1 and 7% for IgG3) [48-50]. The presence of antibodies with a short half-life, as demonstrated in this study, indicates that the IgG3 antibodies against human HSP60 are continuously stimulated and produced. Thus, as suggested in other studies [51,52], the response to human HSP60 seen in SpA patients (Figure 4A) seems to rely on the ability to quickly control and regulate this response. This may explain the decrease in high-level IgG3 against human HSP60 from 2006 to 2010 (Figure 6B) and the average decrease in disease parameters (Table 1). Both human HSP60 and PAD4I are found to be expressed in the synovium, and PAD4I is suggested to form complexes that activate complement proteins, thereby giving rise to the observed correlation between the detection of the protein and the intensity of tissue inflammation in RA [53]. Autoantibodies to human HSP60 may represent a response to a foreign extracellular antigen (that is, mitochondrial HSP60) arising from the ongoing inflammation in the synovial membranes. However, we found no correlation of the antibodies against bacterial and human HSP60 to any of the clinical parameters and MRI scores. Our SpA patients had, in general, low disease activity but a high level of HSP60 antibodies, suggesting that such antibodies did not reflect the disease activity. Nor was there any relation to disease progression in clinical, and MRI scores during the 4-yr observation period. In the present study, HLA-B27 status did not further explain the role of anti-human HSP60, as these antibodies were at the same level in HLA-B27-positive and HLA-B27-negative patients. This is in contrast to findings from a similar study by Dominguez-López *et al*., who investigated the total IgG (total) response to enterobacterial HSP60 in ankylosing spondylitis patients, one of the five disease entities defined within SpA [23]. They found that HLA-B27-positive patients and their healthy relatives had significantly higher IgG antibody levels to all enterobacterial HSP60 proteins than HLA-B27-negative healthy individuals. Furthermore, a correlation between IgG antibodies to *E. coli *and *Shigella flexneri *HSP60 and disease activity was observed in a later study [24]. In these studies, antibodies to human HSP60 were not determined, and patient disease characteristics were not included; therefore, the results cannot be directly compared to those in our study. It should be emphasized that in our study all patients analyzed had axial disease, which does not exclude subjects with concomitant peripheral disease, but it excludes those with only peripheral disease. Therefore data could be different in patients with peripheral SpA.

In summary, we found that IgG3 antibodies against human HSP60 were elevated in SpA patients and that such antibodies with a short half-life were present at both time points separated by more than 4 yr. Therefore, IgG3 against human HSP60 must be produced constantly in these patients, indicating a disease-related function. However, the antibodies were not cross-reactive to bacterial HSP60, indicating that bacteria do not seem to be involved at this stage of disease. Instead, we suggest that the autoantibodies to human HSP60 represent a response to a foreign extracellular antigen (that is, mitochondrial HSP60) arising from the ongoing inflammation in the synovial membranes of these axial SpA patients.

## Conclusions

In a cohort of SpA patients with symptoms restricted to the axial skeleton, elevated levels of IgG1 and IgG3 antibodies to human HSP60 were determined by ELISA. These levels were significantly higher than those in a healthy control group of similar size matched for sex and age. Change in antibody levels differed for IgG1 and IgG3 over time, and there was an inverse relationship of IgG1 to IgG3 antibodies in the sera of the SpA patients. Generation of antibodies to human HSP60 was independent of the presence of antibodies to bacterial HSP60, and cross-reactivity could not be supported from the present study. Finally, we found no evidence that HSP60 antibodies reflected the disease activity in these 39 SpA patients.

## Abbreviations

AS: ankylosing spondylitis; BASDAI: Bath Ankylosing Spondylitis Disease Activity Index; BASFI: Bath Ankylosing Spondylitis Functional Index; BASMI: Bath Ankylosing Spondylitis Metrology Index; CRP: C-reactive protein; ELISA: enzyme-linked immunosorbent assay; ESSG: European Spondyloarthropathy Study Group; ASAS: Assessment of SpondyloArthritis international Society; HC: healthy control; HLA-B27: human leukocyte antigen B27; HSP: heat shock protein; Ig: immunoglobulin; IL: interleukin; IQR: interquartile range; MRI: magnetic resonance imaging; OD: optical density; PsA: psoriatic arthritis; ReA: reactive arthritis; SD: standard deviation; SIJ: sacroiliac joints; SpA: spondyloarthritis; TFI: tubal factor infertility; USpA: undifferentiated spondyloarthritis.

## Competing interests

Svend Birkelund and Gunna Christiansen are shareholders in Loke Diagnostics, Risskov, Denmark, which provided the antigens from *C. pneumonia *and *C. trachomatis *together with the *E. coli *clones used in this study.

## Authors' contributions

TC was responsible for protein purification and SDS gel analysis, designing the serological investigation, acquiring a second serum sample, conducting and analyzing the results and drafting the article. AH contributed to the analysis and interpretation of data and revised the article critically for important intellectual content. BD, AGJ, BSC and AZ contributed to the study's conception and design, the acquisition of consecutive serum samples and analyses of clinical and MRI data, and they revised the article critically for important intellectual content. SB and GC provided the HSP60 clones, contributed to the conception and design of the study and revised the article critically for important intellectual content. All authors read and approved the final manuscript.

## References

[B1] Schlesinger MJ Heat shock proteins J Biol Chem 1990 15 12111 12114 2197269

[B2] De Maio A Extracellular heat shock proteins, cellular export vesicles, and the Stress Observation System: a form of communication during injury, infection, and cell damage Cell Stress Chaperones 2010 15 235 249 20963644 10.1007/s12192-010-0236-4 PMC3077223

[B3] Ritossa FM Experimental activation of specific loci in polytene chromosomes of *Drosophila* Exp Cell Res 1964 15 601 607 10.1016/0014-4827(64)90147-8 14208747

[B4] Stewart GR Young DB Heat-shock proteins and the host-pathogen interaction during bacterial infection Curr Opin Immunol 2004 15 506 510 10.1016/j.coi.2004.05.007 15245747

[B5] Girschick HJ Guilherme L Inman RD Latsch K Rihl M Sherer Y Shoenfeld Y Zeidler H Arienti S Doria A Bacterial triggers and autoimmune rheumatic diseases Clin Exp Rheumatol 2008 15 1 Suppl 48 S12 S17 18570749

[B6] Oldstone MB Molecular mimicry and immune-mediated diseases FASEB J 1998 15 1255 1265 9761770 10.1096/fasebj.12.13.1255 PMC7164021

[B7] Kampinga HH Hageman J Vos MJ Kubota H Tanguay RM Bruford EA Cheetham ME Chen B Hightower LE Guidelines for the nomenclature of the human heat shock proteins Cell Stress Chaperones 2008 15 105 111 18663603 10.1007/s12192-008-0068-7 PMC2673902

[B8] Stevens TR Winrow VR Blake DR Rampton DS Circulating antibodies to heat-shock protein 60 in Crohn's disease and ulcerative colitis Clin Exp Immunol 1992 15 271 274 1424286 10.1111/j.1365-2249.1992.tb07941.x PMC1554622

[B9] Larsen B Birkelund S Mordhorst CH Ejstrup L Andersen LS Christiansen G The humoral immune response to *Chlamydia trachomatis *in patients with acute reactive arthritis Br J Rheumatol 1994 15 534 540 10.1093/rheumatology/33.6.534 7911386

[B10] Handley HH Yu J Yu DT Singh B Gupta RS Vaughan JH Autoantibodies to human heat shock protein (hsp)60 may be induced by *Escherichia coli *groEL Clin Exp Immunol 1996 15 429 435 8608642 10.1111/j.1365-2249.1996.tb08298.x PMC2200362

[B11] Pockley AG Bulmer J Hanks BM Wright BH Identification of human heat shock protein 60 (Hsp60) and anti-Hsp60 antibodies in the peripheral circulation of normal individuals Cell Stress Chaperones 1999 15 29 35 10.1379/1466-1268(1999)004<0029:IOHHSP>2.3.CO;2 10467106 PMC312915

[B12] Anderton SM van der Zee R Prakken B Noordzij A van Eden W Activation of T cells recognizing self 60-kD heat shock protein can protect against experimental arthritis J Exp Med 1995 15 943 952 10.1084/jem.181.3.943 7869052 PMC2191900

[B13] Bodnár N Szekanecz Z Prohászka Z Kemény-Beke Á Némethné-Gyurcsik Z Gulyás K Lakos G Sipka S Szántó S Anti-mutated citrullinated vimentin (anti-MCV) and anti-65 kDa heat shock protein (anti-hsp65): new biomarkers in ankylosing spondylitis Joint Bone Spine 2012 15 63 66 10.1016/j.jbspin.2011.03.010 21683641

[B14] Cappello F Conway de Macario E Di Felice V Zummo G Macario AJL *Chlamydia trachomatis *infection and anti-Hsp60 immunity: the two sides of the coin PLoS Pathog 2009 15 e1000552 10.1371/journal.ppat.1000552 19714222 PMC2726942

[B15] Inman RD Mechanisms of disease: infection and spondyloarthritis Nat Clin Pract Rheumatol 2006 15 163 169 10.1038/ncprheum0118 16932676

[B16] McMichael A Bowness P HLA-B27: natural function and pathogenic role in spondyloarthritis Arthritis Res 2002 15 Suppl 3 S153 S158 10.1186/ar571 12110134 PMC3240147

[B17] Khan MA Mathieu A Sorrentino R Akkoc N The pathogenetic role of HLA-B27 and its subtypes Autoimmun Rev 2007 15 183 189 10.1016/j.autrev.2006.11.003 17289555

[B18] Hammer RE Maika SD Richardson JA Tang JP Taurog JD Spontaneous inflammatory disease in transgenic rats expressing HLA-B27 and human *β*_2_m: an animal model of HLA-B27-associated human disorders Cell 1990 15 1099 1112 10.1016/0092-8674(90)90512-D 2257626

[B19] Taurog JD Richardson JA Croft JT Simmons WA Zhou M Fernández-Sueiro JL Balish E Hammer RE The germfree state prevents development of gut and joint inflammatory disease in HLA-B27 transgenic rats J Exp Med 1994 15 2359 2364 10.1084/jem.180.6.2359 7964509 PMC2191772

[B20] Ekman P Saarinen M He Q Gripenberg-Lerche C Grönberg A Arvilommi H Granfors K HLA-B27-transfected (*Salmonella *permissive) and HLA-A2-transfected (*Salmonella *nonpermissive) human monocytic U937 cells differ in their production of cytokines Infect Immun 2002 15 1609 1614 10.1128/IAI.70.3.1609-1614.2002 11854251 PMC127747

[B21] Ugrinovic S Mertz A Wu P Braun J Sieper J A single nonamer from the *Yersinia *60-kDa heat shock protein is the target of HLA-B27-restricted CTL response in *Yersinia*-induced reactive arthritis J Immunol 1997 15 5715 5723 9548516

[B22] Young JL Smith L Matyszak MK Gaston JSH HLA-B27 expression does not modulate intracellular *Chlamydia trachomatis *infection of cell lines Infect Immun 2001 15 6670 6675 10.1128/IAI.69.11.6670-6675.2001 11598036 PMC100041

[B23] Dominguez-López ML Burgos-Vargas R Galicia-Serrano H Bonilla-Sánchez MT Rangel-Acosta HH Cancino-Diaz ME Jiménez-Zamudio L Granados J García-Latorre E IgG antibodies to enterobacteria 60 kDa heat shock proteins in the sera of HLA-B27 positive ankylosing spondylitis patients Scand J Rheumatol 2002 15 260 265 10.1080/030097402760375133 12455814

[B24] Dominguez-López ML Ortega-Ortega Y Manríquez-Raya JC Burgos-Vargas R Vega-López A García-Latorre E Antibodies against recombinant heat shock proteins of 60 kDa from enterobacteria in the sera and synovial fluid of HLA-B27 positive ankylosing spondylitis patients Clin Exp Rheumatol 2009 15 626 632 19772795

[B25] Hjelholt A Christiansen G Johannesson TG Ingerslev HJ Birkelund S Tubal factor infertility is associated with antibodies against *Chlamydia trachomatis *heat shock protein 60 (HSP60) but not human HSP60 Hum Reprod 2011 15 2069 2076 10.1093/humrep/der167 21642639

[B26] Madsen KB Jurik AG Magnetic resonance imaging grading system for active and chronic spondyloarthritis changes in the sacroiliac joint Arthritis Care Res 2010 15 11 18 10.1002/acr.20008 20191486

[B27] Madsen KB Jurik AG MRI grading method for active and chronic spinal changes in spondyloarthritis Clin Radiol 2010 15 6 14 10.1016/j.crad.2009.08.009 20103415

[B28] Dougados M Van Der Linden S Juhlin R Huitfeldt B Amor B Calin A Cats A Dijkmans B Olivieri I Pasero G Veys E Zeidler H The European Spondylarthropathy Study Group Preliminary Criteria for the Classification of Spondylarthropathy Arthritis Rheum 1991 15 1218 1227 10.1002/art.1780341003 1930310

[B29] Rudwaleit M van der Heijde D Landewe R Listing J Akkoc N Brandt J Braun J Chou CT Collantes-Estevez E Dougados M Huang F Gu J Khan MA Kirazil Y Maksymowych WP Mielants H Sørensen IJ Ozgocmen S Roussou E Weber U Wei J Sieper J The development of Assessment of SpondyloArthritis international Society classification criteria for axial spondyloarthritis (part II): validation and final selection Ann Rheum Dis 2009 15 777 783 10.1136/ard.2009.108233 19297344

[B30] Bowness P HLA B27 in health and disease: a double-edged sword? Rheumatology (Oxford) 2002 15 857 868 10.1093/rheumatology/41.8.857 12154202

[B31] Laemmli UK Cleavage of structural proteins during the assembly of the head of bacteriophage T4 Nature 1970 15 680 685 10.1038/227680a0 5432063

[B32] Schmitt J Hess H Stunnenberg HG Affinity purification of histidine-tagged proteins Mol Biol Rep 1993 15 223 230 10.1007/BF01674434 8114690

[B33] Drasbek M Nielsen PK Persson K Birkelund S Christiansen G Immune response to *Mycoplasma pneumoniae *P1 and P116 in patients with atypical pneumonia analyzed by ELISA BMC Microbiol 2004 15 7 10.1186/1471-2180-4-7 15018643 PMC362870

[B34] Jones CS Maple PAC Andrews NJ Paul ID Caul EO Measurement of IgG antibodies to *Chlamydia trachomatis *by commercial enzyme immunoassays and immunofluorescence in sera from pregnant women and patients with infertility, pelvic inflammatory disease, ectopic pregnancy, and laboratory diagnosed *Chlamydia psittaci*/*Chlamydia pneumoniae *infection J Clin Pathol 2003 15 225 229 10.1136/jcp.56.3.225 12610104 PMC1769898

[B35] Zügel U Kaufmann SH Role of heat shock proteins in protection from and pathogenesis of infectious diseases Clin Microbiol Rev 1999 15 19 39 9880473 10.1128/cmr.12.1.19 PMC88905

[B36] Hoymans VY Bosmans JM Van Renterghem L Mak R Ursi D Wuyts F Vrints CJ Ieven M Importance of methodology in determination of *Chlamydia pneumoniae *seropositivity in healthy subjects and in patients with coronary atherosclerosis J Clin Microbiol 2003 15 4049 4053 10.1128/JCM.41.9.4049-4053.2003 12958224 PMC193860

[B37] Orlik O Ban J Gieciova E Altanerova V Altaner C Two immunodominant regions revealed by monoclonal antibodies on the main structural protein p24 of bovine leukemia virus Viral Immunol 1993 15 245 254 10.1089/vim.1993.6.245 8166932

[B38] Handley HH Yu J Yu DT Singh B Gupta RS Vaughan JH Autoantibodies to human heat shock protein (hsp)60 may be induced by *Escherichia coli *groEL Clin Exp Immunol 1996 15 429 435 8608642 10.1111/j.1365-2249.1996.tb08298.x PMC2200362

[B39] Ethelberg S Mølbak K Olsen K Scheutz F Zoonotic intestinal infections 2008 EPI-NYT National Surveillance of Communicable Diseases 11 2009 15

[B40] Hannu T Reactive arthritis Best Pract Res Clin Rheumatol 2011 15 347 357 10.1016/j.berh.2011.01.018 22100285

[B41] Carter JD Inman RD Whittum-Hudson J Hudson AP *Chlamydia *and chronic arthritis Ann Med 2012 15 784 792 10.3109/07853890.2011.606830 21864020

[B42] Hannu T Puolakkainen M Leirisalo-Repo M *Chlamydia pneumoniae *as a triggering infection in reactive arthritis Rheumatology (Oxford) 1999 15 411 414 10.1093/rheumatology/38.5.411 10371278

[B43] Jefferis R Lund J Pound JD IgG-Fc-mediated effector functions: molecular definition of interaction sites for effector ligands and the role of glycosylation Immunol Rev 1998 15 59 76 10.1111/j.1600-065X.1998.tb01188.x 9700502

[B44] Péne J Gauchat JF Lécart S Drouet E Guglielmi P Boulay V Delwail A Foster D Lecron JC Yssel H Cutting edge: IL-21 is a switch factor for the production of IgG1 and IgG3 by human B cells J Immunol 2004 15 5154 5157 15100251 10.4049/jimmunol.172.9.5154

[B45] Avery DT Bryant VL Ma CS de Waal Malefyt R Tangye SG IL-21-induced isotype switching to IgG and IgA by human naive B cells is differentially regulated by IL-4 J Immunol 2008 15 1767 1779 18641314 10.4049/jimmunol.181.3.1767

[B46] Wang W Li J Predominance of IgG1 and IgG3 subclasses of autoantibodies to peptidylarginine deiminase 4 in rheumatoid arthritis Clin Rheumatol 2011 15 563 567 10.1007/s10067-010-1671-4 21210288

[B47] Adlersberg JB Franklin EC Frangione B Repetitive hinge region sequences in human IgG3: isolation of an 11,000-dalton fragment Proc Natl Acad Sci USA 1975 15 723 727 10.1073/pnas.72.2.723 804697 PMC432388

[B48] Schroeder HW Jr Cavacini L Structure and function of immunoglobulins J Allergy Clin Immunol 2010 15 2 Suppl 2 S41 S52 20176268 10.1016/j.jaci.2009.09.046 PMC3670108

[B49] Papadea C Check IJ Human immunoglobulin G and immunoglobulin G subclasses: biochemical, genetic, and clinical aspects Crit Rev Clin Lab Sci 1989 15 27 58 10.3109/10408368909106589 2647414

[B50] Yount WJ Dorner MM Kunkel HG Kabat EA Studies on human antibodies: VI. Selective variations in subgroup composition and genetic markers J Exp Med 1968 15 633 646 10.1084/jem.127.3.633 4169968 PMC2138455

[B51] van Eden W Wendling U Paul L Prakken B van Kooten P van der Zee R Arthritis protective regulatory potential of self-heat shock protein cross-reactive T cells Cell Stress Chaperones 2000 15 452 457 10.1379/1466-1268(2000)005<0452:APRPOS>2.0.CO;2 11189451 PMC312876

[B52] Quintana FJ Cohen IR HSP60 speaks to the immune system in many voices Novartis Found Symp 2008 15 101 114 137-140 18575269 10.1002/9780470754030.ch8

[B53] Foulquier C Sebbag M Clavel C Chapuy-Regaud S Al Badine R Méchin MC Vincent C Nachat R Yamada M Takahara H Simon M Guerrin M Serre G Peptidyl arginine deiminase type 2 (PAD-2) and PAD-4 but not PAD-1, PAD-3, and PAD-6 are expressed in rheumatoid arthritis synovium in close association with tissue inflammation Arthritis Rheum 2007 15 3541 3553 10.1002/art.22983 17968929

